# Conventional Computed Tomographic Calcium Scoring vs full chest CTCS for lung cancer screening: a cost-effectiveness analysis

**DOI:** 10.1186/s12890-020-01221-8

**Published:** 2020-07-06

**Authors:** Boxiang Jiang, Philip A. Linden, Amit Gupta, Craig Jarrett, Stephanie G. Worrell, Vanessa P. Ho, Yaron Perry, Christopher W. Towe, on behalf of University Hospitals’ Research in Surgical Outcomes and Effectiveness (UH-RISES)

**Affiliations:** 1 Department of Surgery, University Hospitals Cleveland Medical Center, Cleveland, OH USA; 2 Department of Radiology, University Hospitals Cleveland Medical Center, Cleveland, OH USA; 3 Deparment of Trauma and Acute Care Surgery, MetroHealth Hospital, Cleveland, OH USA; 4 University Hospitals Cleveland Medical Center, Division of Thoracic and Esophageal Surgery, 11100 Euclid Avenue, Cleveland, OH 44106-5011 USA

**Keywords:** Lung cancer screening, Conventional computed tomographic calcium scoring, Full chest calcium scoring scan, Cost-effectiveness analysis, Coronary artery disease

## Abstract

**Background:**

Conventional CTCS images the mid/lower chest for coronary artery disease (CAD). Because many CAD patients are also at risk for lung malignancy, CTCS often discovers incidental pulmonary nodules (IPN). CTCS excludes the upper chest, where malignancy is common. Full-chest CTCS (FCT) may be a cost-effective screening tool for IPN.

**Methods:**

A decision tree was created to compare a FCT to CTCS in a hypothetical patient cohort with suspected CAD. (Figure) The design compares the effects of missed cancers on CTCS with the cost of working up non-malignant nodules on FCT. The model was informed by results of the National Lung Screening Trial and literature review, including the rate of malignancy among patients receiving CTCS and the rate of malignancy in upper vs lower portions of the lung. The analysis outcomes are Quality-Adjusted Life Year (QALY) and incremental cost-effectiveness ratio (ICER), which is generally considered beneficial when <$50,000/QALY.

**Results:**

Literature review suggests that rate of IPNs in the upper portion of the lung varied from 47 to 76%. Our model assumed that IPNs occur in upper and lower portions of the lung with equal frequency. The model also assumes an equal malignancy potential in upper lung IPNs despite data that malignancy occurs 61–66% in upper lung fields.

In the base case analysis, a FCT will lead to an increase of 0.03 QALYs comparing to conventional CTCS (14.54 vs 14.51 QALY, respectively), which translates into an QALY increase of 16 days. The associated incremental cost for FCT is $278 ($1027 vs $748, FCT vs CTCS respectively. The incremental cost-effectiveness ratio (ICER) is $10,289/QALY, suggesting significant benefit. Sensitivity analysis shows this benefit increases proportional to the rate of malignancy in upper lung fields.

**Conclusion:**

Conventional CTCS may be a missed opportunity to screen for upper lung field cancers in high risk patients. The ICER of FCT is better than screening for breast cancer screening (mammograms $80 k/QALY) and colon cancer (colonoscopy $6 k/QALY). Prospective studies are appropriate to define protocols for FCT.

## Background

Conventional computed tomographic calcium scoring (CTCS) is a radiographic study frequently used to screen for coronary artery disease (CAD) in asymptomatic patients [1, 2]. Non-cardiac findings are routinely identified on CTCS, including, most commonly, incidental pulmonary nodules (IPN), which are identified in 10–18% of patient [3–5]. Prior studies have shown that work up of these IPNs leads to improved lung cancer mortality [3]. The benefit in lung cancer mortality is limited to the cancers in the lower lung fields. CTCS only images the mid and lower chest and does not include the upper lung fields. Observational studies suggest that pulmonary nodules are more common in the upper lung fields, as primary lung cancers [6, 7]. With this in mind, we believed CTCS may be a missed opportunity to scan the entire lung field for the purposes of identifying more “incidental” lung cancers.

Lung cancer is the leading cause of cancer death in the United States [8]. Based on the success of the National Lung Cancer Screening Trial (NLST) [8], the United States Preventive Service Task Force (USPSTF) recommends screening for lung cancer with low dose computed tomography (LDCT) in patients age 55–80 years who are current smokers (with a 30 pack-year smoking history) or have quit within 15 years [9]. Although lung cancer screening was found to be beneficial in this cohort, it is unclear if there is benefit in other populations.

We hypothesized that adding upper lung field to a calcium scoring test to image the “full chest” (FCT) was more cost-effective than conventional CTCS to screen for CAD and lung cancer. We performed a decision analysis to analyze the potential cost/benefit of the FCT study relative to CTCS.

## Methods

**Fig. 1:**
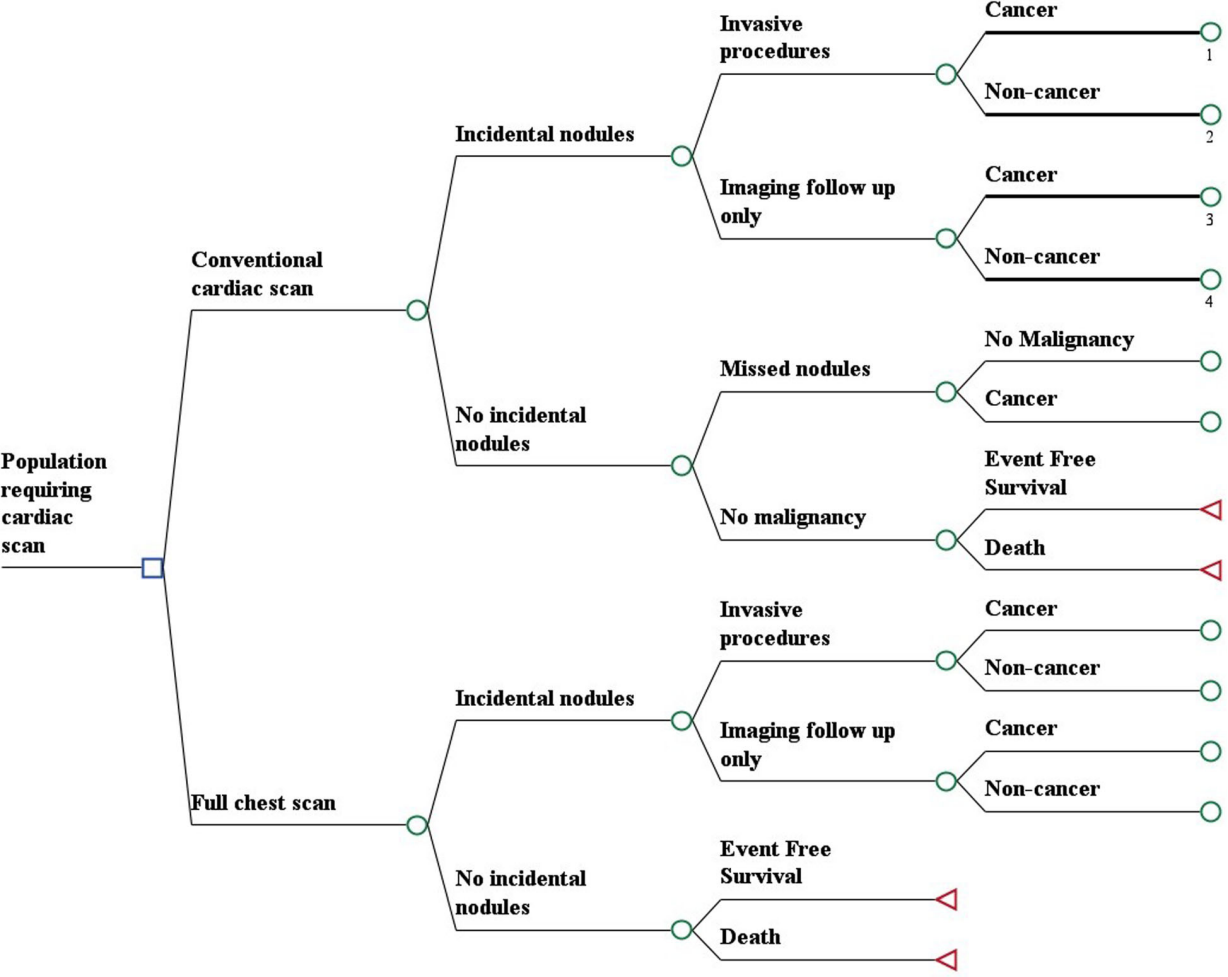
Cost-Effectiveness Analysis Tree-Age Model

A cost-effectiveness decision tree model (Fig. 1) was built using Treeage Pro 2018 (Williamstown, MA). We assumed a hypothetical population with an average age of 50 years who required a screening CTCS to screen for CAD. Patients would either receive a CTCS or FTS as their primary coronary imaging method. The rate of IPN was modeled based on literature review [3–5]. The management of IPNs found on imaging was modeled using data from NLST [8]. IPNs were managed with either invasive procedures for diagnosis or followed by serial imaging. The complications of the invasive procedures were categorized as death, major complications, minor complications, or event free survival. The utility of patients ranges from 0 (death) to 1 (perfect health). A disutility was applied to major and minor complications in the post-procedure periods. If cancer was diagnosed, appropriate cancer treatment was implemented and outcomes were based on NLST data [8]. The outcome of the model was effectiveness and cost. Effectiveness was defined as quality adjusted life year (QALY) and cost reported in dollars ($), and incremental cost-effective ratio (ICER) was calculated from these outcomes.

### Missed pulmonary nodules (MPN)

In CTCS, some pulmonary nodules are not identified due to the limited lung volume included in this imaging modality. For the decision analysis, we assume that the amount of the MPN were proportional to the unscanned lung volume. Because CTCS only includes the lower half of the lung field, we assumed that the amount of MPN were equal to the identified IPNs in CTCS. This may underestimate the true number of IPNs as some studies suggest higher rate of IPNs in the upper lung fields [6, 7]. Despite data regarding the malignant potential of IPN in CTCS [3, 5], the malignancy rate of the MPN is unknown. We assumed that the MPN malignancy rate was the same as the identified IPN, despite some suggestions that lung cancer is more common in the upper lung fields. Patient who received a CTCS and had MPNs were categorized as having either benign disease or malignancy. Those with malignancy were assumed to be present with lung cancer at later stages and received lung cancer treatment.

### Variables

The key variables of the model included the probabilities of incidental nodules in patients receiving CTCS and the rate of malignancy found in these nodules. These variables are shown in Table 1. The rates of invasive procedures and complications from these procedures were modeled from NLST and are also listed.

**Table 1:** Major variables used in the model to analyze the cost effectiveness of full chest CT vs conventional calcium score CT (CTCS)

**Possibilities**	**Base Case**	**Range**	**Reference**
Percentage of patients with IPNs on CTCS	11.25%	N/A	[3]
Percentage of patients with MPNs on CTCS	11.25%	11.25–14.40%	[3–5]
Malignancy rate of IPNs	3.67%	N/A	[3]
Malignancy rate of MPNs	3.67%	3.67–5.5%	[3]
Percentage of IPNs had invasive diagnostic procedure	6.1%	N/A	[8]
Percentage of IPNs followed with imaging only	93.9%	N/A	[8]
Any complications caused by invasive diagnostic procedure	23.9%	N/A	[8]
Major complications after invasive diagnostic procedure	8.1%	N/A	[8]
Mortality caused by invasive diagnostic procedure	1.5%	n/a	[8]
Lifetime cancer recurrence rate	33%	n/a	[10]
**Utility**	**Base Case**	**Range**	**Reference**
Baseline utility	0.845	0.838–0.854	[3, 11–13]
After lung cancer diagnosis	0.62	0.31–0.83	[3, 11–13]
Major complication after invasive procedure	0.5	0.4–0.7	[3, 11, 12]
Minor complication after invasive procedure	0.7	n/a	[3, 11, 12]
**Cost (in 2018 dollar value)**	**Base Case ($)**	**Range ($)**	**Reference**
CTCS	382	156–467	[3]
Invasive procedure	12,321	638–18,970	[11, 12, 14]
Imaging follow-up	954	n/a	[11, 12, 14]
Treating major complications	6524	3262 – 19,678	[11, 12, 14]
Treating minor complications	622	311–933	[11, 12, 14]
Cancer treatment	12,217	6109 – 32,304	[11, 12, 14]

### Base case analysis

The base case analysis represents the best estimation of the “real” difference between patients receiving CTCS and FTS. Results of the base case analysis were effectiveness and cost. A protocol exhibited dominance if it was both life-saving and cost saving. If dominance was not achieved, ICER was calculated. We used ICER of $50,000/QALY as the societal threshold of cost-effectiveness.

### Monte Carlo simulation and sensitivity analysis

A Monte Carlo simulation of 10,000 iterations was performed to test the variability of the model. The major variables were randomly re-sampled in a reasonable range as listed in Table 1. Results are reported as median and 10-90th percentile. Single-variable sensitivity analyses were also performed for percentage of MPNs and malignancy rate of MPNs.

## Results

### Base case analysis

In the base case analysis, FCT is more cost-effective than CTCS, however, FCT does not dominate CTCS. FCT saves an additional 0.03 QALY (14.54 vs 14.51 QALY, FCT vs CTCS respectively) with an additional cost of $278 ($1027 vs $748, FCT vs CTCS respectively). The ICER was calculated to be $10,289/QALY.

### Monte Carlo simulation

A Monte Carlo simulation of 10,000 iterations was performed to assess the variability of the model. The median effectiveness is 14.54 QALY for FCT (14.38 to 14.70, 10th and 90th percentile) compared to 14.51 QALY for CTCS (14.34 to 14.67, 10th and 90th percentile), confirming the benefit found in the base case analysis. The median cost for FCT and CTCS are $1023 and $747, respectively. The median ICER was $10,447/QALY ($8039/QALY to $13,186/QALY, 10th and 90th percentile). These results are summarized in Table 2.

**Table 2:** Monte Carlo Simulation Result of 10,000 iterations

Median [10th - 90th percentile]	CTCS	FCT
Effectiveness (QALY)	14.51 [14.34–14.67]	14.54 [14.38–14.70]
Cost ($)	747 [616–875]	1023 [861–1190]
ICER ($/QALY)	10,447 [8039 – 13,186]	

### Sensitivity analysis

**Fig. 2:**
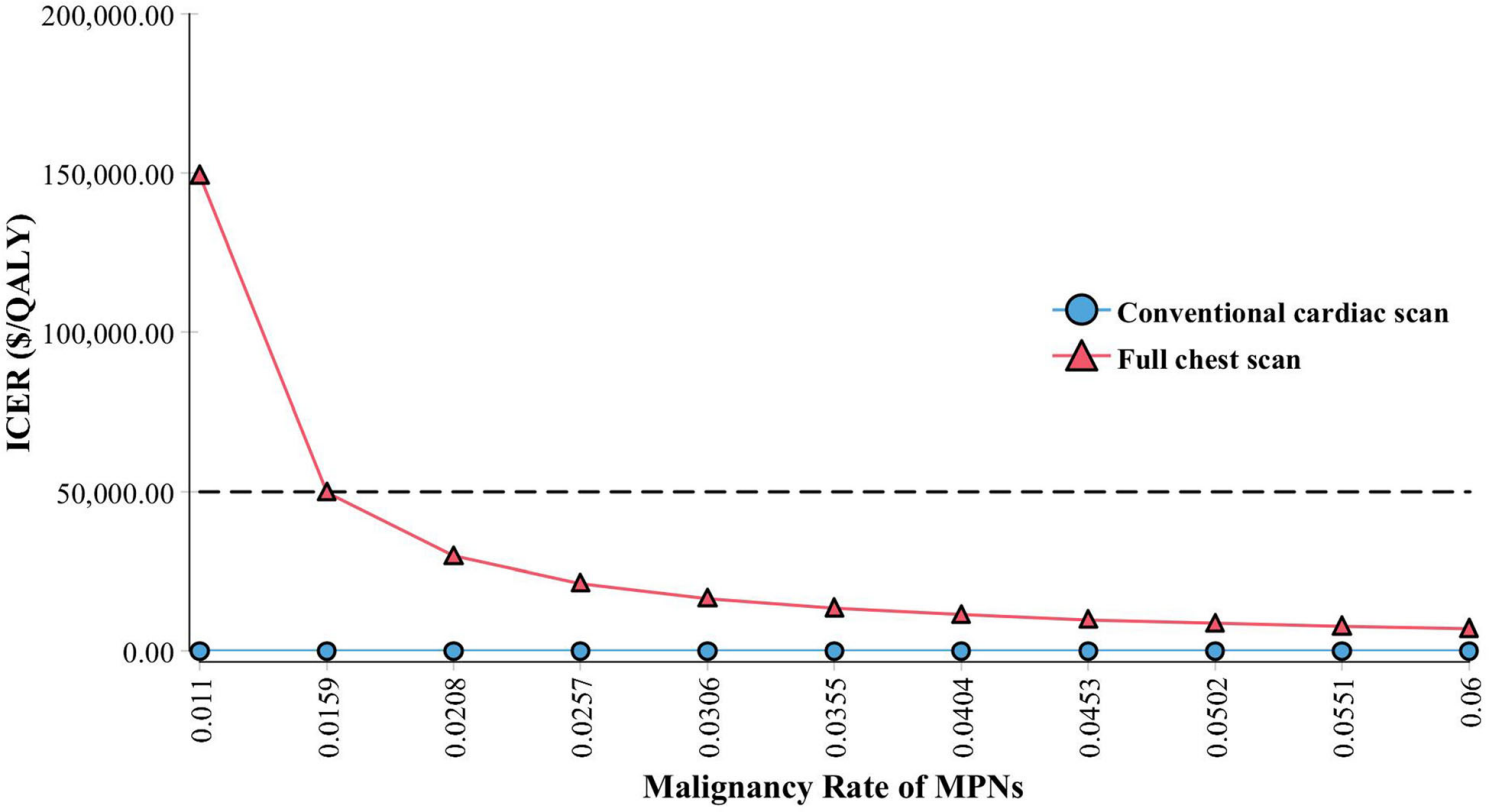
Relationship of malignancy rate of missed pulmonary nodules vs Incremental Cost Effectiveness Ratio (ICER) of “full chest scan” vs conventional calcium score CT (FCT vs CTCS). Dashed line indicates the cost-effective threshold of $50,000/QALY. When the malignancy rate > 1.59%, FCT becomes cost-effective, ie ICER <$50,000/QALY

Sensitivity analyses of the malignancy rate of MPNs were performed. If the malignancy rate of MPNs is higher than 1.59%, FCT becomes more cost-effective than CTCS (ICER < $50,000/QALY). The ICER of FCT decreases with increased malignancy rate of MPNs (Fig. 2). However, the model is not sensitive to the rate of MPNs. The ICER of FCT continues to be at $10,289/QALY when the rate of MPNs varies from 1 to 30%.

## Discussion

In our cost-effectiveness analysis, we showed that a full chest scan is cost-effective in patients requiring CT calcium scoring to screen for coronary artery disease because it saves lives from lung cancer. It saved 0.03 QALY (about 11 days of life) with an additional cost of $278 for a calculated ICER of $10,289/QALY. This result showed FCT was more cost-effective than CTCS. This benefit was further confirmed by Monte-Carlo simulation, which estimated the true ICER to be between $8039/QALY and $13,186/QALY. To put this result into perspective, implementation of mammogram for breast cancer screening has a reported ICER ranging from $60,000 to $80,000/QALY [15]. Screening colonoscopy has an ICER of $15,000/QALY which is comparable to the calculated ICER of FCT [16]. Both screening colonoscopy and mammogram are fully reimbursed by most insurances. While the NLST is currently the “gold standard” study for lung cancer screening, we believe that using the criteria within that study as the only criteria to screen for lung cancer is overly stringent. This study suggests that there is benefit to lung cancer screening in other populations as well. Coronary artery calcification is frequently identified in patients undergoing lung cancer screening with LDCT [17–19]. NLST data have shown that the presence of coronary artery calcification is associated with a 3 fold increase in cardiovascular death [18]. These studies suggest a concordance of CAD and lung cancer [17–19]. Therefore, adding patients who are receiving CTCS to a lung cancer screening cohort is reasonable on several levels.

The actual benefit of FCT is likely underestimated in this study. The actual malignancy rate of the MPNs in this population was unknown, and estimation of this rate was intentionally conservative. Observational studies of pulmonary nodules based on locations suggest that the upper lung fields have more nodules than the lower lung fields and that upper lung nodules tended to have higher malignancy rate [6, 7]. These observational studies were part of our rationale to modify existing CTCS scanning protocols. Interestingly, one study noted that one patient undergoing CTCS developed lung cancer without incidental finding on CTCS suggesting possible missed nodules [4]. In our model, we took a conservative approach in the base-case analysis by assuming an equal malignancy of upper and lower lung field nodules. Even with this assumption, the base-case analysis is cost-effective. Given the conservative nature of our estimates, the actual benefit of FCT may be even more cost-effective than our base-case analysis result. This was corroborated by the sensitivity analysis, which showed that the higher the malignant rate of the MPNs was, the lower the ICER of FCT would become.

Our study has several limitations. Foremost, the patient population who received calcium scoring imaging was different from the NLST inclusion criteria. NLST screened patients aged 55–75 years old who were current smokers or a former smoker who quit less than 15 years prior and with at least 30 pack year smoking history [8]. The criteria for calcium score imaging is not specifically targeted to patient with history of tobacco use. Current expert consensus agreed that calcium score imaging is appropriate for asymptomatic patients who are 40–75 year old with a 5–20% 10 year atherosclerotic cardiovascular disease (ASCVD) risk [2]. The ASCVD risk is calculated based on age, sex, race, smoking status, blood pressure, cholesterol levels, smoking status and diabetic status [2]. Atherosclerotic cardiovascular disease shared risk factors with lung cancer which could explain the benefit of performing FCT on this population [17–19]. Current smoker and former smoker are reported to be around 28 and 39% in patients requiring CTCS [3]. We concede that as tobacco rates decrease, the malignancy rate in IPN will also decrease, thereby erasing the benefit of FCT. While this is a significant limitation, the sensitivity analysis suggests that an FCT protocol for screening coronary artery disease with CT scan will have benefit until the malignancy rate in MPN falls below 1.5%, which is much lower than current estimates [3]. Our model is not sensitive to the amount of the MPNs but only to the malignancy rate of the MPNs. In another word, the cost-effectiveness benefit of FCT derives from identifying malignant pulmonary nodules. As many studies have shown, the majorities of incidental pulmonary nodules found on CTCS are benign [4, 5]. Workup of benign findings will not translate into survival benefit which is proven in our model.

As mentioned above, this study is an exploration of the potential benefit of FCT. In that regard, another weakness of this study is that FCT does not exist as an established imaging protocol. Imaging parameters are yet to be established. Our institution will be creating protocols for these studies and hope to standardize this for international adoption. We included a proposed FCT protocol in the supplemental material (Supplemental Figure 1). Other potential weaknesses of this study are that some outcomes of FCT are not modeled in the outcomes. For example, with increased lung field scanned, more incidental findings such as blebs and emphysematous change may found, and the burden of these findings are not included in the analysis. The increased workload of radiologists was not modelled in our analysis. Radiologists who read CTCS were required to recognize extracardiac findings as these were frequently found on the current CTCS [20]. FCS would identify more extracardiac findings which would require more work of the reading radiologist to characterize these findings. However, we expected the increased workload of FCT would be limited because these extracardiac findings of FCT were similar to that found on CTCS. From a practical standpoint, it would be very difficult to model the increased workload in our current model due to the lack of actual data. We felt it would be better explored in a prospective study. Lastly, the increased radiation exposure to the upper lung fields were not included. Recent studies have shown that the radiation exposure for CTCS is about 1 mSv [21, 22]. Including additional apical lungs for FCT is likely resulting in a negligible increase of overall radiation exposure. Some studies have suggested high radiation exposure in cancer patient cohorts increase the risk of developing cardiovascular disease but the radiation exposure in the study was mainly from radiation therapy instead of diagnostic radiation [23].

The NLST included a strict inclusion criterion to reduce unnecessary screening and interventions. At the same time, it also prevented patients who might harbor lung cancers but did not meet the inclusion criteria from screening and early detection. Patients who were at high risk for lung cancers but did not meet the NLST criteria had a difficult time to obtain a screening LDCT and get insurance to pay for it [24]. This practically excluded them from being screened for lung cancer. Because lung cancer is by far the leading cause of cancer in the United States, we advocate for a new lung screening strategy to combat this disease. With the theoretical benefit for FCT in our study, we recommend implementation of this protocol. Once implemented, prospective study of the true effectiveness and cost could then be realized.

## Conclusion

We concluded in our cost-effectiveness analysis that FCT was more cost-effective than CTCS for screening for lung cancer and coronary artery disease among patients with risk factors for coronary artery disease. This is the first study to show this potential benefit of FCT. Protocols and prospective studies are needed to further establish FCT and realize its full potential.

## Supplementary information

**Additional file 1.** Supplemental Figure 1. Proposed protocol for FCT.

## Data Availability

Not applicable.

## Abbreviations

CAD: Coronary artery disease
CTCS: Conventional computed tomographic calcium scoring
FCT: Full-chest CTCS
ICER: Incremental cost-effectiveness ratio.
IPN: Incidental pulmonary nodules
LDCT: Low dose computed tomography
MPN: Missed pulmonary nodules
NLST: National Lung Cancer Screening Trial
QALY: Quality-adjusted life year
USPSTF: United States Preventive Service Task Force
