# Supplementary material for: Conventional Computed Tomographic Calcium Scoring vs full chest CTCS for lung cancer screening: a cost-effectiveness analysis
Source: BMC Pulm Med. 2020 Jul 6;20:187. doi: 10.1186/s12890-020-01221-8 (PMC7336401; doi:10.1186/s12890-020-01221-8)
Supplement: Supplementary file 1 — Additional file 1. Supplemental Figure 1. Proposed protocol for FCT. [file 12890_2020_1221_MOESM1_ESM.docx]

Supplemental Figure 1. Proposed FCT protocol.

**For Coronary calcium score scan**

**Position:** Supine

**Scanning mode:** Axial with prospective ECG gating

**Scan range:** From below the aortic arch to base of heart

**Scan parameters:** 120 kV fixed, mAs BMI dependent.

BMI < 30 = 120 kVp @ 40 mAs

BMI 31-34 = 120 kVp @ 50 mAs

BMI > 120Kvp @ 60 mAs

**Slice thickness** 2.5 mm with interval of 1 mm

**Reconstructions/ post processing:**

Axial soft tissue widow – Slice thickness – 2.5 mm

Axial lung window- Slice thickness – 2.5 mm

Calcium scoring performed using Philips Intellispace Portal software, Version 10.1,as per Agatson method

**Approximate study time**: 10 – 15 minutes

**For FCT**

**Position:** Supine

**Scanning mode:** Axial with prospective ECG gating

**Scan range:** From base of neck through entire lung fields to base of heart

**Scan parameters:** 120 kV fixed, mAs BMI dependent.

BMI < 30 = 120 kVp @ 40 mAs

BMI 31-34 = 120 kVp @ 50 mAs

BMI > 120Kvp @ 60 mAs

**Slice thickness** 2.5 mm with interval of 1 mm

**Reconstructions/ post processing:**

Axial soft tissue widow – Slice thickness – 2.5 mm

Axial lung window- Slice thickness – 2.5 mm

Calcium scoring performed using Philips Intellispace Portal software, Version 10.1,as per Agatson method

Sagittal and coronal reconstruction

**Approximate study time**: 10 – 15 minutes
